# Residue localization and risk for aspiration in partial laryngectomy: the relevance of assertive therapeutic strategies and resources

**DOI:** 10.31744/einstein_journal/2022AO6262

**Published:** 2022-03-07

**Authors:** Andressa Silva de Freitas, Guilherme Maia Zica, Emilson Queiroz Freitas, Ana Catarina Alves e Silva, Fernando Luiz Dias, Izabella Costa Santos

**Affiliations:** 1 Instituto Nacional de Câncer José de Alencar Gomes da Silva Rio de Janeiro RJ Brazil Instituto Nacional de Câncer José de Alencar Gomes da Silva – INCA - Rio de Janeiro, RJ, Brazil.; 2 Fundação Oswaldo Cruz Rio de Janeiro RJ Brazil Fundação Oswaldo Cruz, Rio de Janeiro, RJ, Brazil.

**Keywords:** Garbage, Deglutition disorders, Laryngectomy, Rehabilitation, Videofluoroscopy, Esophageal sphincter, upper

## Abstract

**Objective:**

To describe the correlation between the residues, their anatomical location and the presence of laryngotracheal penetration and aspiration in patients after supracricoid laryngectomy undergoing cricohyoidoepiglotopexy reconstruction.

**Methods:**

This study included 70 patients treated by supracricoid laryngectomy with cricohyoidoepiglotopexy reconstruction in a referral national cancer hospital. The patients were submitted to swallowing videofluoroscopy, and the findings were classified by the penetration and aspiration scale. The images were described observing the presence or absence of residues and their anatomical location.

**Results:**

The prevalence of penetration in this study was 68.6% and aspiration was 34.3%. An association was found between the presence of residue on the tongue (p=0.005), posterior pharyngeal wall (p=0.013), pyriform recesses (p=0.002), valecula (p=0.061), and laryngeal penetration. The residue in the upper esophageal sphincter (p=0.039) was associated with the occurrence of laryngotracheal aspiration.

**Conclusion:**

Patients undergoing supracricoid laryngectomy with cricohioidoepiglotopexy reconstruction have food residues in different anatomical regions after swallowing. Penetration was associated with the presence of residues on the base of the tongue and posterior pharyngeal wall. Aspiration was associated with the presence of residues in the upper esophageal sphincter.

## INTRODUCTION

Supracricoid laryngectomy (SCPL) is a horizontal partial surgical procedure indicated for T2 to T4 tumors of the glottic and supraglottic region. This procedure main advantage is the preservation of laryngeal functions: laryngeal voice, swallowing and breathing, with absence of permanent stoma.^(1–3)^ The technique consists of removing the lower end of the epiglottis, thyroid cartilage, laryngeal ventricles, vocal folds, ventricular bands, paraglottic space, and preepiglottic space.^(2)^ When neoglottis formation is possible, one or both cricoarytenoid units, the epiglottis and the cricoid, remain. The surgical reconstruction occurs, among variations, by means of the cricohioidoepiglotopexy (CHEP) in which a pexy (suture) is made between the cricoid cartilage, the epiglottis, and the hyoid bone. This reconstruction raises the laryngeal complex to the level of the hyoid bone, leading to a structural rearrangement in the pharyngeal recesses and modification of the swallowing process.^(3–5)^

The larynx is located at the intersection between the upper airway and digestive tract and acts as a sphincter during swallowing to prevent aspiration.^(6)^ After SCPL with CHEP, the patient present dysphagia due to partial loss of this protective mechanism because of the 70% resection of the organ with removal of essential structures, such as vocal folds and vestibular folds. Therefore, justifying the finding of 40% of chronic silent aspiration and residue in different structures (pharyngeal recesses and vallecula) in the late postoperative period of these individuals.^(5,7–10)^

Aspiration is considered as the most relevant finding in swallow imaging studies (videofluoroscopy) because of its potential capacity to cause aspiration pneumonia.^(5,11)^ However, the swallow evaluation is extremely complex, requiring the investigation of other equally relevant aspects such as bolus preparation, transit time, laryngeal penetration, and residue. More specifically regarding residues, their presence, in addition to significantly impairment to quality of life, generates food adaptations and discomfort during feeding, and they may also favor aspiration.^(5,6,8)^

Changing the consistency of food offered orally has become one of the most common forms of intervention for dysphagia. As a therapeutic practice, the principle is to modify the properties of food to make it easier and safer to swallow. In the case of liquids, it is generally agreed that thin liquids (such as water) has safety risks for people with dysphagia because of the greater difficulty in oral motor control and neoglottis closure. For this reason, thickened liquids are often recommended, especially in the immediate postoperative period of SCPL with CHEP in order to slow the transit of liquids and allow more time for airway closure.^(12)^ Maneuvers, strategies and exercises are also widely used by speech pathologists to promote safe and effective swallowing in SCPL with CHEP. Structural, motor and sensory damage from the surgical procedure may impair from bolus transit to upper esophageal sphincter opening.^(5)^

In a deglutition disadvantaged by anatomical modification of the SCPL with CHEP, with impaired sphincter function and increased possibility of residue formation and massive aspiration, it is necessary to use assertive therapeutic resources to promote protection of the lower airways and bolus transit.^(5,8)^ Thus, the motivation for this study was based on the need of understanding the correlation between residues, anatomical locations, and presence of laryngotracheal aspiration. This study sought to improve the speech and hearing management of swallowing disorders in patients undergoing SCPL with CHEP.

## OBJECTIVE

To determine the correlation between pharyngeal residues, their anatomical locations, and the presence of laryngotracheal aspiration in patients who underwent supracricoid laryngectomy with cricohioidoepiglottopexy.

## METHODS

This was a sectional study conducted from January 2015 to December 2017 that included patients with laryngeal cancer treated with SCPL with CHEP enrolled in the Head and Neck Cancer Surgery Section of the *Instituto Nacional de Cancer José Alencar Gomes da Silva* (INCA). The study was approved by the institution's Teaching and Research Committee, CAAE: 26331314.2.0000.5274, # 3892889. All participants who agreed to participate in the research signed a consent form.

The patients were identified by means of their hospital records. We included individuals aged ≥18 years, surgically treated by the technique described by Laccourreye et al.,^(3)^ with no active disease and with at least 6 months of oncologic treatment, and without local recurrence or distant metastases. The patients had no complaints about swallowing, and they were discharged from the speech therapy service, and had no tracheostomy or feeding tube. Adjuvant radiotherapy as part of the treatment protocol was not an exclusion criterion. Patients with cognitive and/or language impairment and those who underwent another type of surgical intervention in the head and neck region before or after SCPL with CHEP were excluded.

Swallowing videofluoroscopy was used to objectively evaluate the swallowing of the study volunteers. Videofluoroscopic examinations were performed at the Radiology Department of Inca, according to the protocol based on the study by Logemann^(13)^ and adapted at the *Laboratório Interdisciplinar de Cabeça e Pescoço* (LICEP).^(7)^

The Penetration Aspiration Scale (PAS) developed by Rosenbek et al., was utilized as a parameter to analyze the presence and absence of penetration, as well as the aspiration at the swallowing videofluoroscopy. According to Rosenbek et al., penetration is defined as the passage of the bolus to the laryngeal level, without passing below the vocal folds. Aspiration is defined as the passage of the material below the level of the vocal folds.^(14)^ In this study, we used the neoglottis level as the reference structure for penetration, as it is the last barrier for laryngotracheal aspiration.

The descriptive analysis, on the distribution of demographic and clinical characteristics of the study population, was presented by means of frequency measures for categorical variables. The χ² test and Fisher's exact test were adopted. To analyze the independent variables and their association with the outcomes.

The outcomes were categorized as present or absent, presenting two separate models at the end. The statistical modeling used was logistic regression, and each independent variable was tested by a bivariate approach, obtaining as a crude measure of association the odds ratio (OR). The variables were selected for the multiple models according to the criteria of theoretical relevance and statistical significance (Wald test). The stepwise method was used to include the variables in the multiple model and select the final model. For all statistics cited here, a significance level of 5% (<0.05) was considered. All analyses were performed in the R for Windows program, version 3.6.1.

## RESULTS

We evaluated 70 patients, predominantly elderly, aged over 60 years (80%). Most of them were men (98.6%), married (75.7%), and with low formal education (54.3%). More than 80% of the individuals were smokers, and at least 60% reported alcohol consumption at the time of diagnosis. Adding the categories single, divorced, and widowed, we obtained 24.3% (n=17) who reported no partner at the time of data collection. As for staging, we observed proportions of 38.6% and 32.9% among individuals staged II and III.

Nasogastric tube and tracheostomy removal occurred within 45 days in 54.3% and 65.7%, respectively. Radiotherapy was performed in just over 30% of patients.

The distribution of sociodemographic data and clinical information of the individuals in the evaluated group are shown in table 1.

**Table 1:** Clinical and sociodemographic information

Variables		n (%)
Sex		
	Men	69 (98.6)
	Women	1 (1.4)
Age, years		
	<60	14 (20)
	60-69	25 (35.7)
	70-79	27 (38.6)
	≥80	4 (5.7)
Marital status		
	Married	53 (75.7)
	Single	8 (11.4)
	Divorced	3 (4.3)
	Widowed	6 (8.6)
Education		
	Illiterate	2 (2.9)
	Primary school	48 (54.3)
	High school	25 (35.7)
	Higher education	5 (7.1)
	Smoker	57 (81.4)
	Alcohol consumption	46 (65.7)
Clinical staging		
	I	19 (27.1)
	II	27 (38.6)
	III	23 (32.9)
	IV	1 (1.4)
Nasogastric tube, days		
	≤45	38 (54.3)
	46-89	20 (28.6)
	>90	12 (17.1)
Tracheostomy, days		
	≤45	46 (65.7)
	46-89	12 (17.1)
	>90	12 (17.1)
	Radiotherapy	22 (31.9)
Arytenoids		
	1	32 (47.7)
	2	38 (54.3)

During the objective examination of swallowing, the prevalence was 68.6% of laryngeal penetration and 34.3% of laryngotracheal aspiration.

Table 2 describes the simple and multiple logistic regression of the relationship of the anatomical region of residue after swallowing and the presence of laryngeal penetration and laryngotracheal aspiration of the 70 individuals of the study.

**Table 2:** Simple and multiple logistic regression for anatomical region of residue

Residue		Penetration				Aspiration			
Anatomic region		Odds ratio (95% CI)*	p value	Odds ratio (95%CI)†	p value	Odds ratio (95%CI)*	p value	Odds ratio (95%CI)†	p value
Base of the tongue									
	No	1		1		1			
	Yes	10.73 (2.31-77.54)	0.005	17.91 (1.59-3.08)	0.027	1.05 (0.25-5.36)	0.949		
Posterior wall of pharynx									
	No	1		1		1			
	Yes	13.76 (2.54-256.82)	0.013	7.31 (1.13-147)	0.078	1.42 (0.47-4.14)	0.525		
Vallecula									
	No	1				1			
	Yes	3.27 (0.94-11.71)	0.061			0.80 (0.23-2.95)	0.726		
Upper esophageal sphincter									
	No	1				1		1	
	Yes	1.70 (0.37-12.16)	0.527			4.78 (1.13-24.65)	0.039	5.35 (0.94-44.75)	0.075
Piriform recesses									
	No	1				1			
	Yes	7.17 (2.13-27.01)	0.002			1.57 (0.46-6.26)	0.485		

* Simple logistic regression raw odds ratio;

† multiple logistic regression adjusted odds ratio final predictive model; controlled for age and number of arytenoids.

OR: odds ratio; 95%CI: 95% confidence interval.

Individuals with residue on the base of the tongue after swallowing had a 17 times greater chance of laryngeal penetration than those without residue in this location. Individuals with residues on the posterior pharyngeal wall and piriform recesses had a 7 times greater chance of laryngeal penetration than those without residues in these regions. Individuals with residues in the vallecula after swallowing had a 3 times greater chance of laryngeal penetration than individuals without residues.

Laryngeal penetration was associated with the presence of residues on the base of the tongue and posterior wall of the pharynx.

In addition, individuals with residues in the upper esophageal sphincter after swallowing were 5 times more likely to have laryngotracheal aspiration than individuals without residues in this anatomical region.

Laryngotracheal aspiration was associated with the presence of residues in the upper esophageal sphincter.

## DISCUSSION

In the specialized literature, there is evidence of functional complications after SCPL with CHEP with regard to breathing and swallowing functions.^(2,6)^ It is known that episodes of penetration and/or aspiration may occur occasionally throughout the life of some patients. In this group there is also a major impairment of the lower airway protection structures that may favor the presence of chronic aspiration even in the absence of complaint.^(5,7)^ However, due to the complexity of assessing this aspect, there is a scarcity of studies seeking to understand the consequences and signs from this chronic aspiration.^(4,7,15,16)^

Historically, specialized literature has focused on laryngotracheal aspiration, with little relevance on food residues and their consequences. The presence of residues often causes more impact than aspiration in quality of life.^(8,12)^

Swallowing function has been widely discussed in SCPL with CHEP over the past 10 years. However, to date, methods have mainly addressed aspiration findings, with little investigation addressing other parameters, such as pharyngeal residues. Pizzorni et al. reported the importance of a broader evaluation of swallowing and, nevertheless, categorize the instrument used in two items (safe and not safe), without investigating the impact of residues in their results.^(17)^

No national studies were found about residues in patients undergoing SCPL with CHEP who, despite good oncological control and return of exclusive oral diet, showed a large number of residues after swallowing in the evaluated group. Furthermore, it is possible to assume that there is a direct correlation between residue locations and the chances of occurrence of laryngeal penetration and laryngotracheal aspiration.

In the present study, the tongue base was the most prevalent region in residue formation and correlated with the presence of laryngeal penetration. This functional finding may mean a change in swallowing biodynamics, as a result of surgical reconstruction, with traction of the tongue base through the hyoid bone and cricoid cartilage pexis, due to the CHEP,^(18)^ which connects the muscles of the floor of the mouth, tongue, larynx, epiglottis and pharynx.^(19)^ The contact between the base of the tongue and the posterior wall of the pharynx is important in applying the pressure required for ejection of the bolus and its transport through the pharynx into the esophagus.^(20)^ Logemann et al. stated that the contact of the tongue base with the posterior wall of the pharynx at the time of ejection is a critical factor in swallowing recovery after partial laryngectomies.^(6)^ It is possible to understand that the new anatomical organization promotes a *deficit* in food bolus ejection due to the reduced tongue mobility and therefore favors residue formation.^(10)^ Moreover, recent studies stated that individuals over 60 years of age have a predisposition to residues at the base of the tongue and vallecula, resulting from presbyphagia (reduced muscle mobility and calcification of the epiglottis).^(21)^

The base of the tongue generates pressure to propel the bolus through the oropharynx during swallowing.^(22)^ The rapid, posterior movement of the base of the tongue toward the posterior wall of the pharynx favors bolus ejection and pharyngeal clearing.^(23)^ Reddy et al. found a correlation between tongue strength and clearing capacity, *i.e*., percentage of residue.^(24)^

The findings showed that laryngeal penetration was associated with the presence of residue on the posterior pharyngeal wall. After the onset of pharyngeal swallowing, the anterior movement of the posterior pharyngeal wall starts at the level of the upper oropharynx and, anatomically, the upper, middle and lower pharyngeal constrictor muscles are responsible for the sequential contraction of the pharyngeal wall. The posterior wall, together with the lateral pharyngeal walls, move medially and eventually meet the base of the tongue, which moves posteriorly, during the ejection of the bolus. The contact of the posterior pharyngeal wall with the base of the tongue results in a pressure on the crushed food, forcing it downward, and the pharyngeal contraction progresses downward to the esophageal inlet, following the tail of the bolus.^(6,22,24)^ The structural modification after resection and pexis in SCPL with CHEP promotes an impact on food bolus ejection and, consequently, a pharyngeal contraction, sometimes insufficient and not modulated to its volume and viscosity, favoring residue formation in posterior pharyngeal wall.

The residue in the vallecula was associated with penetration in this series. This finding may be justified by the change in mobility of the epiglottis resulting from resection of its petiole, its deposition with the cricoid cartilage and hyoid bone, and its cicatricial process.^(3,6,18)^ In addition, the already described tongue base changes add up to difficulties in eversion of the epiglottis due to its surgical fixation, favoring residue formation, and the presence of penetration after swallowing.

The surgical technique described by Laccourreye et al. includes myotomy of the inferior pharyngeal constrictor muscle. Therefore, the region of the new hypopharynx has less mucosal support by the remaining structure, which creates a food retention area in the pharyngoesophageal transition and piriform recesses. Laccourreye et al. recommended repositioning the piriform recess during the surgical procedure, in an attempt to maintain the food path with smaller regions favorable to pharyngeal residues.^(1,3)^

In this study, the only structure with positive correlation for the presence of aspiration was the residue in the upper esophageal sphincter. Such finding may be justified by the impaired propulsion of the bolus by pharyngeal contraction and subsequent hyolaryngeal excursion. These biomechanics should favor the opening of the upper esophageal sphincter in the amplitude and time necessary for complete passage of the bolus and avoid the presence of residues. It is known that the surgical technique for SCPL with CHEP includes myotomy of the inferior pharyngeal constrictor muscle, a structure that, with the anterior movement of the larynx, should pull and allow relaxation of the upper esophageal sphincter.^(1,3,5,9)^

The swallowing sequelae of patients submitted to SCPL with CHEP are chronic, ^i.e.^, due to the resection of a large part of the laryngeal valvular structures and the restructuring of the entire hyolaryngeal complex, the oropharyngeal dynamics no longer conforms to the normality standard. That is the patient starts having a high risk of aspiration, and this may have frequent episodes, and there is a high chance of residue formation due to structural changes, and their functional consequences.^(5,7,8,15)^ Rehabilitation is essential to adapt the patient to the new swallowing biomechanics, aiming at reducing the damage caused by resection of the structures, and their consequent reconstruction.^(5,25)^ It is important to emphasize that, although SCPL with CHEP directly affects the pharyngeal phase of swallowing, functional repercussions (mobility, vigor and duration) are observed in all its stages. Specific speech language pathology techniques, such as vocal exercises, tongue exercises and other structures that make up the oral phase, in addition to swallowing facilitating maneuvers, need to be used as important strategies for the development of compensations.^(5)^

The tongue after SCPL with CHEP has a significant reduction in its mobility and coordination. Therefore, strategies need to be used immediately after the procedure and the stability of the condition, to enable a functional swallow, with greater safety and efficacy.^(5,8,12)^ In the therapeutic process, tongue exercises, such as counter-resistance, lateralization and tongue rotation in the vestibule, are fundamental for a better functional result, due to the compromised ejection and transit of the bolus.^(5,7,15,21)^

The Shaker et al. technique is a strengthening exercise that aims to promote greater opening of the upper esophageal sphincter, favoring transit of the bolus by stimulating the suprahyoid muscles. This technique can be a relevant resource in an attempt to reduce residues in pharyngeal recesses and bolus sensation. However it is necessary to consider important factors of general health to ensure safety during the exercise.^(26,27)^

Regarding maneuvers, the supraglottic and supersupraglottic maneuvers allow greater range of motion and longer duration of neoglottis closure during swallowing. This resource favors airway protection in patients with swallowing alterations in the immediate post-operative period or even in chronic individuals with laryngeal penetrations and silent aspirations.^(5,28)^

The deglutition with effort and Masako maneuver favor contact between the tongue base and the posterior pharyngeal wall, which may enable better bolus propulsion and the consequent gain in swallowing dynamics.^(5,6,28)^ Additionally, these strategies favor pharyngeal clearing, which reduces the amount of residue and the chances of penetration and aspiration after swallowing.^(8)^

It is already a consensus that incomplete laryngeal closure affects swallowing safety, leading to laryngotracheal penetration and aspiration.^(5,9)^ Vocal exercises such as thrusting may be beneficial for neoglottis coaptation and airway protection, as well as vibratory and semi-occluded tract exercises with different cervical postures and movements.^(29)^

This study had some limitations. A differential analysis of swallowing performance with stratification of different consistencies and volumes was not performed. It is possible to infer that these aspects may also influence swallowing dynamics. The residues were not classified using standardized scales for objective examinations of swallowing. Despite understanding the importance of therapeutic strategies, their efficacy was not evaluated in this population. Further studies with intervention groups should be conducted to describe the impact of these strategies in the future.

Because of the major impairments in swallowing dynamics from the surgical procedure, patients undergoing SCPL with CHEP have food residues in different anatomical structures after swallowing. This aspect favors the presence of penetration and aspiration, even in individuals without complaints. Swallowing therapy and its various strategies are essential at all times of treatment to ensure better functional results and maintenance of quality of life, potentially increasing survival.^(5,8)^

## CONCLUSION

Patients undergoing supracricoid laryngectomy with cricohioidoepiglottopexy present food residues in different anatomical regions after swallowing. The base of the tongue, posterior pharyngeal wall, and vallecula were locations with food residue accumulation. This fact positively correlated with the presence of penetration. The esophageal sphincter was the area of residue accumulation with the greatest risk of aspiration. The speech therapist must plan his/her therapy aiming at compensating the *deficits* coming from the surgical procedure and the maintenance of the quality of life, within the possibilities of the new anatomy.
